# Correction: Multimorbidity and food insecurity in adults: A systematic review and meta-analysis

**DOI:** 10.1371/journal.pone.0304247

**Published:** 2024-05-20

**Authors:** Maria Kantilafti, Konstantinos Giannakou, Stavri Chrysostomou

In the Author Contributions, Konstantinos Giannakou should be listed as one of the persons who wrote the paper.
