# Supplementary material for: Multimorbidity and food insecurity in adults: A systematic review and meta-analysis
Source: PLoS One. 2023 Jul 6;18(7):e0288063. doi: 10.1371/journal.pone.0288063 (PMC10325088; doi:10.1371/journal.pone.0288063)
Supplement: S2 Table — (DOCX) [file pone.0288063.s002.docx]

**Supplementary Table 2:** Summary of the search strategy

|  | **Key Words** | **PubMed**  **(Title/ Abstract)** | **EBSCO**  **(Abstract)** | **Scopus**  **(Title-Abs_Key)** |
| --- | --- | --- | --- | --- |
| **#1** | “Food Supply” OR “Food Supplies” OR “food insecurity” OR “food insecurities” OR “food insecure” OR “food deficit” OR “lack of food” OR “food scarcity” OR “food shortage” OR “food uncertainty” OR “food security” OR “food secure” OR “food insufficiency*” | 23527 | 20298 | 98924 |
| **#2** | Multimorbid* OR Multi-morbid* OR comorbid* OR co-morbid* OR “multiple chronic conditions” OR “multiple conditions” OR “multiple illnesses” OR “multiple diseases” OR “multiple diagnoses” OR “morbidity pattern” OR “morbidity patterns” OR polymorbidity OR “poly-morbidity” OR “poly morbidity” OR polymorbidities OR “poly-morbidities” OR “poly morbidities” OR multipatholog* OR “multi-pathology” OR “multi pathology” | 255375 | 244266 | 461627 |
| **#1 AND #2** | | 168 | 152 | 239 |
