# Supplementary figures and images for: Multimorbidity and food insecurity in adults: A systematic review and meta-analysis
Source: PLoS One. 2023 Jul 6;18(7):e0288063. doi: 10.1371/journal.pone.0288063 (PMC10325088; doi:10.1371/journal.pone.0288063)

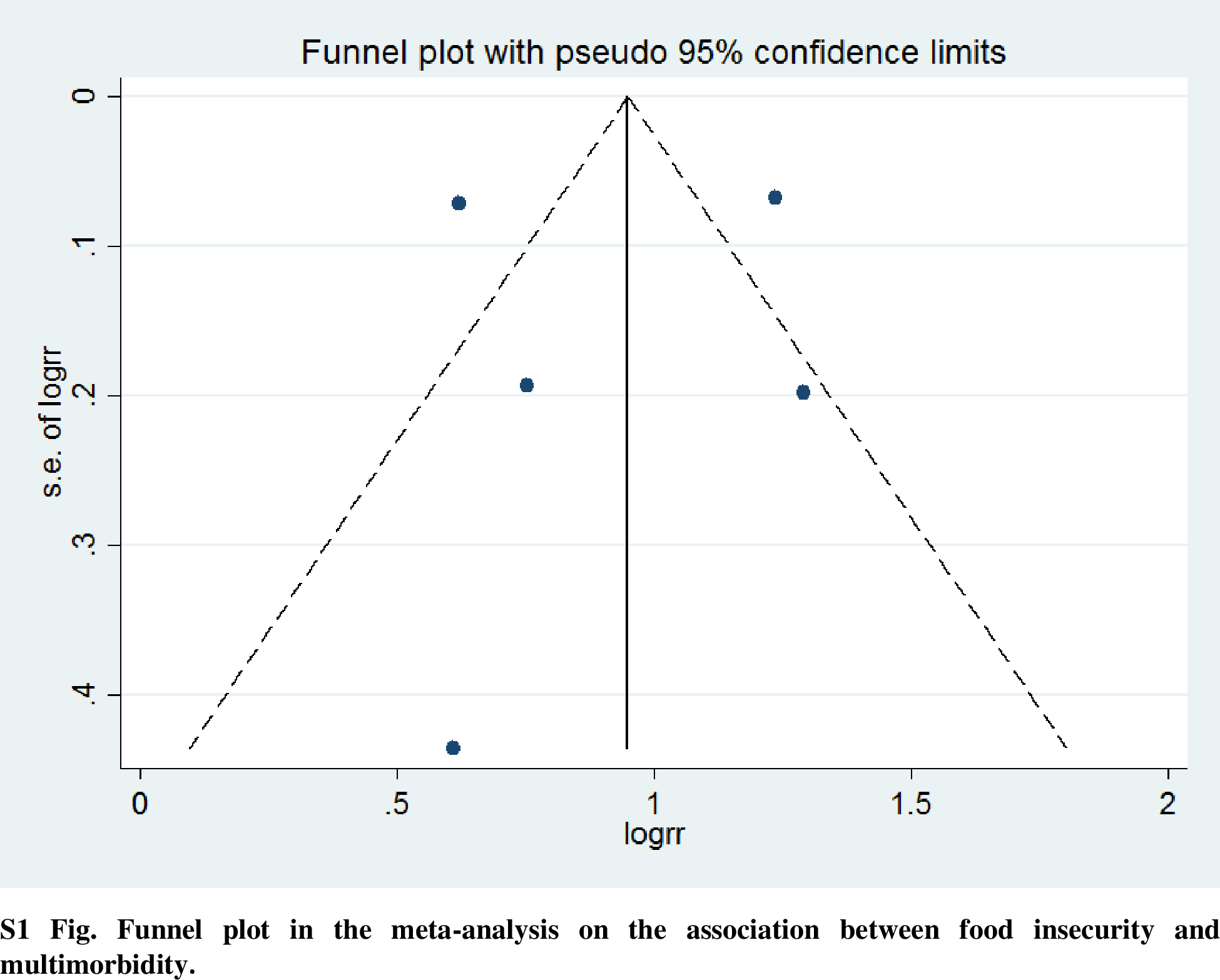

Supplement: S1 Fig — (TIF) [file pone.0288063.s003.tif]

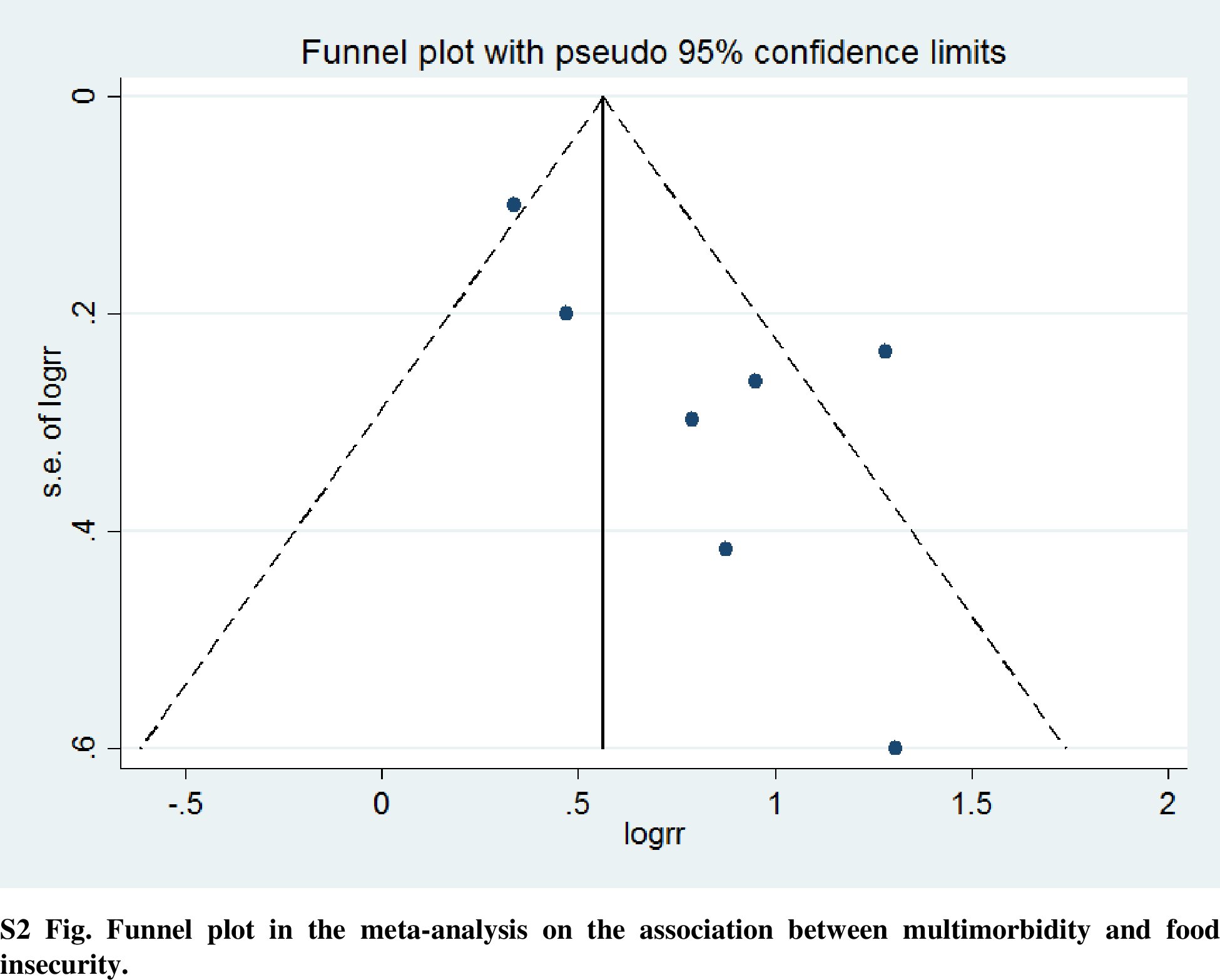

Supplement: S2 Fig — (TIF) [file pone.0288063.s004.tif]
