# Supplementary material for: Multimorbidity and food insecurity in adults: A systematic review and meta-analysis
Source: PLoS One. 2023 Jul 6;18(7):e0288063. doi: 10.1371/journal.pone.0288063 (PMC10325088; doi:10.1371/journal.pone.0288063)
Supplement: Attachment — Submitted filename: PONE-D-22-34406.pdf [file pone.0288063.s006.pdf]

PLOS ONE

Food Insecurity and multimorbidity in adult population: A systematic review and meta-analysis

--Manuscript Draft--

**Manuscript Number:**	PONE-D-22-34406
**Article Type:**	Research Article
**Full Title:**	Food Insecurity and multimorbidity in adult population: A systematic review and meta-analysis
**Short Title:**	Food insecurity and multimorbidity
**Corresponding Author:**	Stavri Chrysostomou, PhD<br>European University Cyprus School of Sciences<br>Nicosia, CYPRUS
**Keywords:**	Food insecurity, multimorbidity, food insecure, chronic diseases, meta-analysis
**Abstract:**	Background<br>If you start by defining multimorbidity, the title could be modified based on it. like Food insecurity could come after multimorbidity Or Food insecurity could come first in the background<br>Multimorbidity is considered to be a global public health challenge and it is affected by several factors. Food insecurity is one potential factor associated with multimorbidity with researchers supporting the possible reverse causation between them. It must be noted that there is a scarcity of evidence regarding the abovementioned association. Thus, the purpose of this systematic review and meta-analysis is to examine the relationship between food insecurity and multimorbidity in adults.<br><br>Methods<br><br>A systematic literature review of studies was performed using the PubMed, EBSCO and SCOPUS for all articles published in English until December 2022. Two independent reviewers screened all retrieved articles that were likely to meet the inclusion criteria. Meta-analysis was performed considering results from the fully adjusted model. The methodological quality was assessed using the Newcastle-Ottawa Scale adapted for cross-sectional studies.<br>The range could come from ---to---- and the starting year was missed<br><br>Results<br>Our? Better to say the study findings showed ....<br><br>Seven studies involving almost 128,000 adults > 18 years of age were included in this meta-analysis. Four cross-sectional studies involving 45,404 participants were included in order to investigate the possible impact that food insecurity has on multimorbidity. Our findings showed an increased probability of multimorbidity 1.55 (95% CI:1.31-1.79, $p<0.001$) among people with food insecurity. Conversely, three of the included studies, involving 81,080 participants concluded that people with multimorbidity, have 2.38 (95% CI: 1.69-3.07, $p<0.001$, $I2 = 92.4\%$) times higher odds to present food insecurity.<br><br>Conclusion<br><br>This systematic review and meta-analysis provide evidence of reverse association between food insecurity and multimorbidity. Further studies are required in order to elucidate further the association of multimorbidity and food insecurity.
**Order of Authors:**	Stavri Chrysostomou, PhD<br>Further study suggestion is not clearly described here
	Maria Kantilafti
	Konstantinos Giannakou
**Additional Information:**	
**Question**	**Response**
**Financial Disclosure**	NO

Enter a financial disclosure statement that describes the sources of funding for the work included in this submission. Review the submission guidelines for detailed requirements. View published research articles from *PLOS ONE* for specific examples.<br><br>This statement is required for submission and **will appear in the published article** if the submission is accepted. Please make sure it is accurate.<br><br>**Unfunded studies**<br>Enter: *The author(s) received no specific funding for this work.*<br><br>**Funded studies**<br>Enter a statement with the following details:<br>• Initials of the authors who received each award<br>• Grant numbers awarded to each author<br>• The full name of each funder<br>• URL of each funder website<br>• Did the sponsors or funders play any role in the study design, data collection and analysis, decision to publish, or preparation of the manuscript?<br>• **NO** - Include this sentence at the end of your statement: *The funders had no role in study design, data collection and analysis, decision to publish, or preparation of the manuscript.*<br>• **YES** - Specify the role(s) played.<br><br>* typeset	
**Competing Interests**<br><br>Use the instructions below to enter a competing interest statement for this submission. On behalf of all authors, disclose any competing interests that could be perceived to bias this work—acknowledging all financial support and any other relevant financial or non-financial competing interests.<br><br>This statement is required for submission and **will appear in the published article** if the submission is accepted. Please make	NO AUTHORS HAVE COMPETING INTERESTS

sure it is accurate and that any funding sources listed in your Funding Information later in the submission form are also declared in your Financial Disclosure statement.<br><br>View published research articles from *PLOS ONE* for specific examples.<br><br>**NO authors have competing interests**<br><br>Enter: *The authors have declared that no competing interests exist.*<br><br>**Authors with competing interests**<br><br>Enter competing interest details beginning with this statement:<br><br>*I have read the journal's policy and the authors of this manuscript have the following competing interests: [insert competing interests here]*<br><br>* typeset	
**Ethics Statement**<br><br>Enter an ethics statement for this submission. This statement is required if the study involved:<br><br>• Human participants<br>• Human specimens or tissue<br>• Vertebrate animals or cephalopods<br>• Vertebrate embryos or tissues<br>• Field research<br><br>Write "N/A" if the submission does not require an ethics statement.<br><br>General guidance is provided below. Consult the submission guidelines for detailed instructions. **Make sure that all information entered here is included in the Methods section of the manuscript.**	N/A

**Format for specific study types**<br><br>**Human Subject Research (involving human participants and/or tissue)**<br>• Give the name of the institutional review board or ethics committee that approved the study<br>• Include the approval number and/or a statement indicating approval of this research<br>• Indicate the form of consent obtained (written/oral) or the reason that consent was not obtained (e.g. the data were analyzed anonymously)<br><br>**Animal Research (involving vertebrate animals, embryos or tissues)**<br>• Provide the name of the Institutional Animal Care and Use Committee (IACUC) or other relevant ethics board that reviewed the study protocol, and indicate whether they approved this research or granted a formal waiver of ethical approval<br>• Include an approval number if one was obtained<br>• If the study involved *non-human primates*, add *additional details* about animal welfare and steps taken to ameliorate suffering<br>• If anesthesia, euthanasia, or any kind of animal sacrifice is part of the study, include briefly which substances and/or methods were applied<br><br>**Field Research**<br><br>Include the following details if this study involves the collection of plant, animal, or other materials from a natural setting:<br>• Field permit number<br>• Name of the institution or relevant body that granted permission	
**Data Availability**<br><br>Authors are required to make all data underlying the findings described fully available, without restriction, and from the time of publication. PLOS allows rare exceptions to address legal and ethical concerns. See the PLOS Data Policy and FAQ for detailed information.	Yes - all data are fully available without restriction

A Data Availability Statement describing where the data can be found is required at submission. Your answers to this question constitute the Data Availability Statement and **will be published in the article**, if accepted.<br><br>**Important:** Stating 'data available on request from the author' is not sufficient. If your data are only available upon request, select 'No' for the first question and explain your exceptional situation in the text box.<br><br>Do the authors confirm that all data underlying the findings described in their manuscript are fully available without restriction?	
**Describe where the data may be found in full sentences. If you are copying our sample text, replace any instances of XXX with the appropriate details.**<br><br>• If the data are **held or will be held in a public repository**, include URLs, accession numbers or DOIs. If this information will only be available after acceptance, indicate this by ticking the box below. For example: *All XXX files are available from the XXX database (accession number(s) XXX, XXX.).*<br>• If the data are all contained **within the manuscript and/or Supporting Information files**, enter the following: *All relevant data are within the manuscript and its Supporting Information files.*<br>• If neither of these applies but you are able to provide **details of access elsewhere**, with or without limitations, please do so. For example:<br><br>*Data cannot be shared publicly because of [XXX]. Data are available from the XXX Institutional Data Access / Ethics Committee (contact via XXX) for researchers who meet the criteria for access to confidential data.*<br><br>*The data underlying the results presented in the study are available from (include the name of the third party*	All relevant data are within the manuscript and its Supporting Information files

and contact information or URL).<br>• This text is appropriate if the data are owned by a third party and authors do not have permission to share the data.<br><br>* typeset	
Additional data availability information:	

Manuscript Click here to access/download;Manuscript;Manuscript.docx

Food Insecurity and multimorbidity in adult population: A systematic review and meta-analysis

Maria Kantilafti[1], Konstantinos Giannakou[1], Stavri Chrysostomou[2]

[1] Department of Health Sciences, School of Sciences, European University Cyprus, Nicosia, Cyprus
[2] Department of Life Sciences, School of Sciences, European University Cyprus, Nicosia, Cyprus

Short title: Food insecurity and multimorbidity

Correspondence: Stavri Chrysostomou, PhD, Department of Life Sciences, European University Cyprus, Nicosia, Cyprus, 6 Diogenes Str. Engomi, 2404, P.O. Box: 22006, 1516, Nicosia, Cyprus. Email:

Abstract

The abstract section could end in not more than one page and could capture all the required information for readers. The structured abstract could remove the subtopic and the order will tell readers as it was written in the form of structured format

Background

Multimorbidity is considered to be a global public health challenge and it is affected by several factors. Food insecurity is one potential factor associated with multimorbidity with researchers supporting the possible reverse causation between them. It must be noted that there is a scarcity of evidence regarding the abovementioned association. Thus, the purpose of this systematic review and meta-analysis is to examine the relationship between food insecurity and multimorbidity in adults.

The background information lacks in-text citation. the background is not adequate and not supported by quantitative evidence

Methods

A systematic literature review of studies was performed using the PubMed, EBSCO and SCOPUS for all articles published in English until December 2022. Two independent reviewers screened all retrieved articles that were likely to meet the inclusion criteria. Meta-analysis was performed

considering results from the fully adjusted model. The methodological quality was assessed using the Newcastle-Ottawa Scale adapted for cross-sectional studies.

Results

Seven studies involving almost 128,000 adults > 18 years of age were included in this meta-analysis. Four cross-sectional studies involving 45,404 participants were included in order to investigate the possible impact that food insecurity has on multimorbidity. Our findings showed an increased probability of multimorbidity 1.55 (95% CI:1.31-1.79, $p<0.001$) among people with food insecurity. Conversely, three of the included studies, involving 81,080 participants concluded that people with multimorbidity, have 2.38 (95% CI: 1.69-3.07, $p<0.001$, $I^2 = 92.4\%$) times higher odds to present food insecurity.

Conclusion

This systematic review and meta-analysis provide evidence of reverse association between food insecurity and multimorbidity. Further studies are required in order to elucidate further the association of multimorbidity and food insecurity.

Introduction

Multimorbidity is a term commonly used to define the coexistence of at least two chronic conditions in the same individual (Fox et al, 2007). It is estimated that the prevalence of multimorbidity is increasing through the decades, regardless of the individual's race and ethnicity (Caraballo, et al., 2022). It is even more common in the elderly (Fortin, et al., 2012), with researchers supporting the claim that prevalence of multimorbidity among the general population older than 75 years is over 80% (Pefoyo, et al., 2015). At the same time, it has been found that, in

absolute numbers, there are more people with multimorbidity who are younger than 65 years, based on the fact that those people represent the larger segment of the population (Barnett, et al., 2012). Based on the above and keeping in mind that multimorbidity has an impact on a person's quality of life, along with other consequences like high health care costs, disability (McPhail, 2016), premature death (Gijsen, et al., 2001) and mortality (Menotti, et al., 2001), multimorbidity must be considered a global public health challenge.

Several factors have been identified that contribute to multimorbidity, including age (Fabbri, et al., 2015), a low educational level (Nagel, et al., 2008), living in a deprived area (Pathirana et al, 2018), low levels of physical activity (Delpino, et al., 2022), and unhealthy lifestyle (Nagel, et al., 2008). Another potential factor that has been reported is food insecurity, with some researchers supporting the possible reverse causation between multimorbidity and food insecurity (Tarasuk, et al., 2013). Food insecurity is the result of limited or inadequate access to nutritionally and safely sound food, due to individual factors such as low income, mobility limitations, and other factors such as limited access to grocery stores or transportation barriers (Barrett, 2010). On one hand, food insecurity may have a negative impact on a person's quality of life affecting both mental and physical health (Summit, 1996 ; Farrell, et al., 2018 ; Moradi, et al., 2018). Many researchers support the potential relation between food insecurity and chronic diseases including obesity (Moradi, et al., 2018), diabetes mellitus (Gucciardi, et al., 2009), hypertension (Mendy, et al., 2018) and cancer (Gany, et al., 2014) but there is a scarcity of evidence regarding the possible relation between food insecurity and multimorbidity. On the other hand, people with multiple chronic conditions may be unable to work, thus having an unstable income. Furthermore, having multiple chronic conditions increases expenditures for medications, physiotherapy, and transportation to doctors (Tarasuk, et al., 2013). Based on the above, multimorbidity may cause an

economic burden on individuals, resulting in food insecurity. As both food insecurity and multimorbidity are characterized with high prevalence (Koné Pefoyo, et al., 2015 ; FAO, IFAD, UNICEF, 2021) and also have an impact on a person's quality of life, identifying an association between them could contribute to more targeted programs and policies.

To the best of our knowledge, this is the first systematic review and meta-analysis to investigate the association among food insecurity and multimorbidity. Thus, the purpose of this systematic review and meta-analysis is to investigate the relationship between food insecurity and multimorbidity in adults, as well as vice versa. Exploring this gap and measuring the impact that food insecurity has on multimorbidity on the one hand and the impact that multimorbidity has on food insecurity on the other would be extremely important for public health actions and programs related to multimorbidity and food insecurity.

What will be the policy implication of this study ??

Methods

The method is not adequate. The authors could show all the main procedures followed during the data gathering /review and meta analysis

The systematic review is conducted in accordance with the Preferred Reporting Items for Systematic Reviews and Meta‐Analysis (PRISMA) (Moher, et al., 2009). The PRISMA 2009 checklist is shown in (S1 Table).

Search strategy and eligibility criteria

A systematic literature search was conducted through the international databases, including PubMed, EBSCO and Scopus using Medical Subject Headings (MeSH) terms. The references cited in the selected articles were also searched manually. The literature search was conducted independently by two researchers (MK and KG) from inception to December 2022. The search was limited to papers published in English; there was no date restriction on the studies included.

Where the literature search was stared?

Any discrepancies between reviewers were resolved in consultation with a third author (SC). The search strategy used is presented in (S2 Table).

To identify included studies, we used the Participants, Interventions, Comparisons, Outcomes, and Study Design (PICOS) method. Participants were ≥ 18-year-old adults. Multimorbidity was considered both the intervention/exposure and the outcome of interest, defined as the presence of at least two chronic conditions in the same individual (Fox et al, 2007). For the comparison, we used participants with 0-1 chronic conditions. The outcome of interest as well as exposure was food insecurity, which is defined as the result of limited or inadequate access to nutritionally and safe food (Barrett, 2010). Any study design was eligible for inclusion. We did not include studies conducted with samples of institutionalized adults (e.g., hospitalized or residents of nursing homes). Published conference abstracts, dissertations, narrative reviews, and case reports were also excluded.

as described by Fox et al(2007)

Any study design was eligible for inclusion ??? this is not clear and the text is standalone. Exclusion criterion was not clearly justified

Study selection and data extraction

You can stop to explain about the two reviewers and better to explain the search process

The titles and abstracts from the electronic searches were examined independently by two reviewers (MK and KG) and the full-text articles from the selected studies that were likely to meet the inclusion criteria were retrieved. Following the first stage, the same authors contacted a second evaluator in order to make the final decision. Any discrepancies between reviewers were resolved in consultation with a third author (SC). From each study, we extracted the following variables: author, year, and country; study design and population; main measures; multimorbidity definition and prevalence, food insecurity definition and prevalence; comparison group; multimorbidity and food insecurity assessment as well as confounders assessed.

The study extracted ....i , we types of explanation is not encouraged in academic publication

Quality assessment

Cross sectional and two reviewers? Has not prelateships plus avoid explaining the reviewers again Directly go to the point and describe the quality assurance methods

All selected studies were cross-sectional and therefore, two reviewers (MK and KG) assessed the methodological quality of each selected study using the Newcastle-Ottawa quality assessment scale adapted for cross-sectional studies (Modesti, et al., 2016). The Newcastle-Ottawa scale assesses the quality of the studies in three sectors, with the maximum number of stars awarded being ten: 1) the selection of the study group (representativeness of the sample, sample size, non-responders, and ascertainment of the exposure) scores up to five stars, 2) the comparability of subjects in different outcome groups on the basis of design or analysis scores up to two stars, and finally 3) the aspects of the outcome such as the assessment of the outcome and the statistical test score up to three stars. “Very good studies” and “good studies” were defined as those that scored nine or ten points and seven or eight points, respectively. Studies with five or six stars were categorized as satisfactory, and studies with less than four points were categorized as unsatisfactory. Any disagreement was resolved by discussion with a third author [SC].

Statistical Analysis

the setence is too long

The association between food security and multimorbidity and vice versa was assessed using the odds ratios (OR) and corresponding 95% confidence intervals (CI) across the studies included. For the meta-analysis, we considered results from the fully adjusted model. We assessed heterogeneity

between variables not studies

between studies, and we reported the P-value of the χ^2-based Cochran Q test and the I^2 metric for inconsistency (26). If $I^2 > 50\%$, a random effects model was used; otherwise, a fixed-effect model was applied (27). The publication bias was examined by visual inspection of funnel plots and evaluated formally with Egger’s regression asymmetry test (29, 30). All statistical analyses were performed by STATA 14.0 software (STATA Corp, College Station, TX), and a two-tailed P value <0.05 was deemed statistically significant.

Results

Description of studies

The inclusion criterion could be explained before discussing the the excluded papers

The flow chart of selection studies regarding the systematic review is presented in Fig 1. From the initial search, 559 articles were identified out which 302 duplicate records were removed. Out of 257 articles which were screened based on their title and abstract, 206 articles were excluded as they did not meet the required inclusion criteria. From the remaining 51 articles, 8 articles were included for qualitative analysis (Jih, et al., 2020; Jih, et al., 2018; Sharkey, 2003; Fernandes, et al., 2018; Smith, et al., 2022; Leung, et al., 2020; Tarasuk, et al., 2013; O'neal, et al., 2022) whereas 7 articles were enrolled in the quantitative meta-analysis (Jih, et al., 2020; Jih, et al., 2018; Sharkey, 2003; Smith, et al., 2022; Leung, et al., 2020;Tarasuk, et al., 2013; O'neal, et al., 2022) (Fig 1).

This subsection looks like methodology and requires revision

Study characteristics of the studies included

The exclusion and inlusion is already described in the methods of analysis

The characteristics of the included studies in the qualitative synthesis which defined multimorbidity or food insecurity as the outcome of interest are presented in **Table 1.** See the language and revise More than 45,000 adults > 18 years of age were included in the systematic review in order to examine the potential effect that food insecurity has on multimorbidity. All studies included employed a cross-sectional design, were published between 2003 and 2022, and contacted in United States (Sharkey, 2003; Leung, et al., 2020; O'neal, et al., 2022) while one study was conducted in many countries (Smith, et al., 2022). Food insecurity was measured using the 6-item (Leung, et al., 2020) or the 18-item US Household Food Security Survey Module (O'neal, et al., 2022), two questions about food insecurity (Smith, et al., 2022), and four self-reported food insecurity risk situations questions (Sharkey, 2003). In all studies included the presence of

multimorbidity was assessed using self-reported chronic conditions questionnaires. Data collection was performed using a face-to-face interview (Smith, et al., 2022; O'neal, et al., 2022; Sharkey, 2003) or a computer in order to complete an online questionnaire (Leung, et al., 2020).

The citation doesnot show your effort. It seems you are talking about what other authors did and no one can understand it.

For the investigation of the potential impact that multimorbidity has on food insecurity, we included three studies with almost 83, 000 adults > 18 years of age All cross-sectional studies, were published between 2013 and 2020, and contacted in United States (Jih, et al., 2018; Jih, et al., 2020), Canada (Tarasuk, et al., 2013) and Portugal (Fernandes, et al., 2018). Multimorbidity, was assessed using self-reported chronic conditions questionnaires. Food insecurity, the outcome of interest, was measured using the 6-item (Jih, et al., 2018) or the 18-item US Household Food Security Survey Module (Tarasuk, et al., 2013), the 10-item US Adult Food Security Survey Module (Jih, et al., 2020) and a psychometric scale adapted from the Brazilian Insecurity Scale (Fernandes, et al., 2018). Regarding the data collection, researchers used different methods such as a computer-assisted telephone interview method (Fernandes, et al., 2018; Jih, et al., 2020) a US mail service (Jih, et al., 2018), or a combination of methods (Tarasuk, et al., 2013).

Quality Assessment and Publication Bias

Discuss the results and cite the Table. Just use economy of words across the paper. Again the application of in text citation is wrong. Most of the studies

Table 3 presents the results of the quality assessment. Most of the studies had scored 5 out of 10, classified as "satisfactory studies" (Leung, et al., 2020; Sharkey, 2003; Smith, et al., 2022; O'neal, et al., 2022; Fernandes, et al., 2018; Tarasuk, et al., 2013). The remaining two studies by Jih et al, had scored 4 out of 10 indicating "unsatisfactory" studies (Jih, et al., 2018; Jih, et al., 2020). The publication bias of the studies was determined by visual inspection of funnel plots for asymmetry or outliers and then evaluated formally with Egger's regression asymmetry test. The funnel plots showed reasonable symmetry, with no evidence of publication bias. Likewise, no evidence of publication bias was observed according to Egger asymmetry test ($P_{Egger's\ test}$ P > 0.05) except for

Most of the texts deserved to be described at the methodology section

studies on the association between food insecurity and multimorbidity ($P_{Egger's\ test}$ <0.05) (S1 and 2 Fig).

The effect of food insecurity on multimorbidity

The same problem. This is result section not methodology.

Among the eight studies included in the qualitative analysis, four of them met the eligibility criteria to be included in the meta-analysis for examining the effect of food insecurity on multimorbidity. Using the fixed effect model, we found that people with food insecurity have 1.55 (95% CI: 1.31-1.79, p<0.001) times higher odds for multimorbidity. There was no significant statistical heterogeneity among study results (I^2 = 44.1%, p=0.097) (Fig 2).

The effect of multimorbidity on food insecurity

The same problem mixing methods and results

Among the eight studies included in the qualitative analysis, three of them met the eligibility criteria to be included in the meta-analysis for examining the effect of multimorbidity on food insecurity as the outcome of interest. A random-effect model was applied to analyze the data and compared to people without multimorbidity (0-1 chronic conditions), those who present ≥ 2 chronic conditions have 2.38 (95% CI: 1.69-3.07, p<0.001, I^2 = 92.4%) times higher odds for food insecurity (Fig 3). The results of the study that was not included in the meta-analysis (Fernandes, et al., 2018) is presented in Table 1.

Discussion

This sis not the appropriate location to discuss on the problem statement/gaps in the previouse studies

To the best of our knowledge, this is the first systematic review and meta-analysis that examine the bidirectional association between food insecurity and multimorbidity. Four cross-sectional studies involving 45,404 participants were included in order to investigate the possible effect that

food insecurity has on multimorbidity. Our findings suggested that, compared to being food secure, food insecurity was associated with 1.55 (95% CI:1.31-1.79) times higher odds for multimorbidity. Conversely, three of the included studies examined the possible association between multimorbidity and food insecurity, and we found that people with multimorbidity have 2.38 (95% CI:1.69-3.07) times higher odds of experiencing food insecurity.

The discussion section is supposed to describe the results with the support of previous related studies on the major variables relationship with adequate authors implication on the results

The findings of our meta-analysis are broadly in line with two previous studies on this topic. A data analysis form the National Health and Nutrition Examination Survey for the years 2007-2014 found that food insecure obese adults were more likely to have more comorbidities conditions than food secure obese adults (Palakshappa, et al., 2019). Another study, conducted in Portugal, concluded that food-insecure households had greater odds of having one or more comorbidities (Fernandes, et al., 2018). To date, there is no other study examining the association between food insecurity and multimorbidity.

There is problem of in text citation across the paper.

It is worth mentioning that among factors associated with multimorbidity (Nagel, et al., 2008; Pathirana et al, 2018; Delpino, et al., 2022), age seems to have an impact on this global challenge (Fortin, et al., 2012). Based on the latest data from United Nations, worldwide, life expectancy, reached 72.8 years in 2019, showing an increase of almost 9 years since 1990. Further reductions in mortality will lead to even more increase in life expectancy reaching 77.2 years in 2050 (Gaigbe-Togbe, et al., 2022). According to WHO data, the proportion of the global population aged 60 years and older will almost double from 12% to 22% between 2015 and 2050 (World Health Organization, 2018). Based on the above and keeping in mind that multimorbidity is more common among the elderly population, it is obvious that health systems will have to face an increasing number of people living with multimorbidity (Nunes, et al., 2016). Furthermore, it is worth mentioning that the prevalence of multimorbidity in populations is difficult to be measured,

due to the use of different definitions and classifications. For example, a systematic review of 39 observational studies across 12 countries reported the prevalence of multimorbidity to be between 13% and 95%. Although most of the studies were defined multimorbidity as the presence of at least 2 chronic diseases in the same individual there were studies that considered multimorbidity as the presence of at least 3 chronic diseases (Violan, et al., 2014). Another systematic review reported similar results with multimorbidity varying from 3.5% to 98.5% in primary care patients and between 12% and 71.8% among the general population. According to researchers, the most important factor responsible for the above variation is the definition of multimorbidity, with some studies to defined multimorbidity as having 2 or more chronic conditions and other to defined multimorbidity as the presence of 3 or 4 chronic conditions (Fortin, et al., 2012). Notably, recent data by FAO support that the prevalence of food insecurity has also increased from 22.6% in 2014 to 29.3% in 2021 globally. Regarding Europe, 8.0% of the population are food insecure with 1.5% of them being severe food insecure (FAO, 2022). Therefore, taking into account the significant number of people suffering from food insecurity on one hand and the continues increase in the prevalence of multimorbidity on the other hand, further studies examining the association among food insecurity and multimorbidity are emerged.

Citation? for another study

If you say according to researchers, you have to cite immediately not end of the texts

If so, where is the value added of this paper? If your study result is similar to other previous related studies?? It is obviouse that food insecurity has a lot of health problems

In order to investigate the potential mechanisms for the association between food insecurity and multimorbidity we cannot exclude the possibility of reverse causation (Tarasuk, et al., 2013). On one hand, controlling multimorbidity requires additional expenditures for medications, physiotherapy, transportation and doctor's visits. As a results of the above, people with multimorbidity face an additional strain on the household budget leading to an increased risk of developing food insecurity (Tarasuk, et al., 2013 ; Valtorta, et al, 2013). At the same time, multimorbidity can affect adults ability to garner income in order to minimize experiences of food

insecurity due to their physical and mental limitations (Tarasuk, et al., 2013). On the other hand, due to lack of money, food insecure people are characterized with increased consumption of unhealthy food which is typically cheaper and more readily available than healthy alternatives leading to obesity and other obesity-related diseases (Rosas, et al., 2011; Jafari, et al., 2017; Gubert, et al., 2016; Eckel, et al., 2014). Moreover, based on previous research, it seems that food insecurity is ever more severe among low-income population. Particularly, a previous study in Cyprus indicated that families would need to spend up to two-thirds of their monthly income to eat healthily (Chrysostomou et al., 2017). Thus, a poor diet due to food insecurity may lead to inadequate nutritional and antioxidant status. As a result of that, people present a systematic low-grade inflammation reflected by elevated C-reactive protein (CRP) levels (González-Fernández, et al., 2017; Zhou, et al., 2022). Therefore, inflammation may be a mechanism by which food insecurity leads to multiple chronic diseases including heart disease, hypertension and diabetes mellitus (Vozoris, et al., 2003; Mendy, et al., 2018).

Notably, food insecurity is associated with unhealthy body weight including both obesity and underweight. source? As for obesity, food insecure people consume cheaper but high energy density foods leading to a much higher energy intake compared to energy expenditure. Therefore, food insecure people are in a high risk of developing obesity and the relative adverse outcomes (Mendis, et al, 2011). Based on the above, food insecurity could lead to multimorbidity through obesity given that obesity is the main leading cause of many chronic non-communicable diseases (Safaei, et al., 2021). Regarding being underweight, food insecurity may lead to inadequate food consumption due to financial barriers which can cause negative energy balance and undernourishment (Gowda, et al., 2012). A recent study suggest that people with BMI < 18 kg/m^2 were significantly more likely to suffer from five or more chronic conditions (Cwirlej-Sozanska, et al., 2020). Therefore,

unhealthy body weight could be the key factor for the association between food insecurity and multimorbidity and thus, controlling body weight could eliminate both conditions.

It's worth mentioning that food insecurity could also result to poor chronic disease management. For example, in regards to diabetes, food insecure people with diabetes report poor glycemic control because of increased difficulty in following a diabetic nutritional therapy and emotional distress due to their weakness for successful diabetes self-management (Seligman, et al., 2012). Based on the literature, uncontrolled diabetes may lead to other chronic diseases such as kidney diseases and other cardiovascular diseases (Kazancioğlu, 2013). Moreover, recent studies aimed to examine the risk of experiencing food insecurity among people suffering from chronic diseases. In particular, a study in Cyprus has shown that the Cypriot gluten-free Healthy diet is costly and not affordable among low-income Cypriots diagnosed with celiac disease, and thus, patients are likely to experience food insecurity, compromising their long-term health (Chrysostomou, et al., 2020). In contrast, regarding to diabetes, the same researchers found that the diabetic nutritional therapy is more affordable compared to other diets such as the healthy diet or the gluten free healthy diet (Chrysostomou, et al., 2020). Therefore, it could be assumed that not all chronic diseases lead to food insecurity. It is emerged to conduct more studies to identify the risk of food insecurity caused by each chronic disease and even more in multimorbidity.

The current study has some limitations. Despite the effort to find all eligible articles on this subject, gray literature and studies published in language other than English were not included in our search. In addition, we excluded a study from the meta-analysis due to the number of comorbidities found in a continuous form (Fernandes, et al., 2018). Another limitation is the fact that some studies used different questionnaires in order to evaluate food insecurity which may restrict their comparability. Finally, most of the studies evaluated multimorbidity using self-report

methods which may increase the risk of recall bias. However, it is worth mentioning that self-report method for multimorbidity can provide reliable results compared to data originating from primary health care system records (Violán, et al., 2013).

Conclusions

This systematic review and meta-analysis provide evidence of reverse association between food insecurity and multimorbidity. Food insecure people have 1.55 higher odds of presenting multimorbidity. At the same time people with multimorbidity have 2.38 higher odds to present food insecurity. Our findings emphasize the need for further research in order to better clarify the determinants of multimorbidity and food insecurity. Furthermore, more studies are needed to increase comparability between them and reach to more accurate results. Considering the above, it is expected that public health policies should implement strategies assessing food insecurity especially for people who are at high risk being food insecure and multimorbidity in order to develop successful public health programs for controlling multimorbidity and food insecurity.

Author contributions

The policy recommendation is too general and needs specific recommendation

MK conceived the theme, conducted the literature search, assessed the articles, summarized the results, prepared the tables, wrote the manuscript, and interpreted the results. KG conducted the literature search, performed the meta-analysis, and interpreted the results. SC supervised the process and interpreted the results. All authors contributed to the article and approved the submitted version.

Funding

This research received no specific grant from any funding agency.

Conflict of interest

The authors declared no potential conflicts of interest.

Bibliography

Bibliography and reference is two different things. I suggest to say references

Barnett, K., Mercer, S. W., Norbury, M., Watt, G., Wyke, S., & Guthrie, B. (2012). Epidemiology of multimorbidity and implications for health care, research, and medical education: A cross-sectional study. *The Lancet*. https://doi.org/10.1016/S0140-6736(12)60240-2

Barrett, C. B. (2010). Measuring food insecurity. *Science*. https://doi.org/10.1126/science.1182768

Caraballo, C., Herrin, J., Mahajan, S., Massey, D., Lu, Y., Ndumele, C. D., Drye, E. E., & Krumholz, H. M. (2022). Temporal Trends in Racial and Ethnic Disparities in Multimorbidity Prevalence in the United States, 1999-2018. *American Journal of Medicine*, *135*(9), 1083-1092.e14. https://doi.org/10.1016/j.amjmed.2022.04.010

Chrysostomou, S., Andreou, S. N., & Andreou, C. (2020). The development of the gluten free healthy food basket in Cyprus. Is it affordable among low-income adults diagnosed with celiac disease? *Journal of Public Health (United Kingdom)*. https://doi.org/10.1093/pubmed/fdz034

Chrysostomou, Stavri, & Andreou, S. (2017). Do low-income Cypriots experience food stress? The cost of a healthy food basket relative to guaranteed minimum income in Nicosia, Cyprus. *Nutrition and Dietetics*. https://doi.org/10.1111/1747-0080.12322

Chrysostomou, Stavri, Koutsampelas, C., Andreou, S. N., & Pittas, C. (2020). The purchase of

the Diabetic Healthy Food Basket in Cyprus results in cost savings: Is it affordable among the low-income population? *Public Health Nutrition*. https://doi.org/10.1017/S1368980020001184

Cwirlej-Sozanska, A. B., Wisniowska-Szurlej, A., Wilmowska-Pietruszynska, A., Sozanski, B., & Mazur, A. (2020). Effect of body weight on disability and chronic disease rates in the elderly in south-eastern poland. *Annals of Agricultural and Environmental Medicine*. https://doi.org/10.26444/aaem/108542

Delpino, F. M., de Lima, A. P. M., da Silva, B. G. C., Nunes, B. P., Caputo, E. L., & Bielemann, R. M. (2022). Physical Activity and Multimorbidity Among Community-Dwelling Older Adults: A Systematic Review With Meta-Analysis. *American Journal of Health Promotion*, *0*(0), 1–15. https://doi.org/10.1177/08901171221104458

Eckel, R. H., Jakicic, J. M., Ard, J. D., De Jesus, J. M., Houston Miller, N., Hubbard, V. S., Lee, I. M., Lichtenstein, A. H., Loria, C. M., Millen, B. E., Nonas, C. A., Sacks, F. M., Smith, S. C., Svetkey, L. P., Wadden, T. A., & Yanovski, S. Z. (2014). 2013 AHA/ACC guideline on lifestyle management to reduce cardiovascular risk: A report of the American college of cardiology/American heart association task force on practice guidelines. *Journal of the American College of Cardiology*. https://doi.org/10.1016/j.jacc.2013.11.003

Fabbri, E., An, Y., Zoli, M., Simonsick, E. M., Guralnik, J. M., Bandinelli, S., Boyd, C. M., & Ferrucci, L. (2015). Aging and the burden of multimorbidity: Associations with inflammatory and anabolic hormonal biomarkers. *Journals of Gerontology - Series A Biological Sciences and Medical Sciences*. https://doi.org/10.1093/gerona/glu127

FAO, IFAD, UNICEF, W. and W. (2021). The State of Food Security and Nutrition in the World 2021. Transforming food systems for food security, improved nutrition and affordable

healthy diets for all. Rome, FAO. https://doi.org/https://doi.org/10.4060/cb4474en

FAO. (2022). *The State of Food Insecurity and Nutrition in the World*. Retrieved from https://www.fao.org/3/cc0639en/cc0639en.pdf

Farrell, P., Thow, A. M., Abimbola, S., Faruqui, N., & Negin, J. (2018). How food insecurity could lead to obesity in LMICs. *Health Promotion International*. https://doi.org/10.1093/heapro/dax026

Fernandes, S. G., Rodrigues, A. M., Nunes, C., Santos, O., Gregório, M. J., de Sousa, R. D., Dias, S., & Canhão, H. (2018). Food insecurity in older adults: Results from the epidemiology of chronic diseases cohort study 3. *Frontiers in Medicine*. https://doi.org/10.3389/fmed.2018.00203

Fortin, M., Stewart, M., Poitras, M. E., Almirall, J., & Maddocks, H. (2012). A systematic review of prevalence studies on multimorbidity: Toward a more uniform methodology. *Annals of Family Medicine*. https://doi.org/10.1370/afm.1337

Fox, R., & Fletcher, J. (2007). Alarm symptoms in primary care. *British Medical Journal*. https://doi.org/10.1136/bmj.39212.467037.BE

Gaigbe-Togbe, V., Bassarsky, L., Gu, D., Spoorenberg, T., & Zeifman, L. (2022). *World Population Prospects 2022*. Retrieved from https://www.un.org/development/desa/pd/sites/www.un.org.development.desa.pd/files/wpp2022_summary_of_results.pdf

Gany, F., Lee, T., Ramirez, J., Massie, D., Moran, A., Crist, M., McNish, T., Winkel, G., & Leng, J. C. F. (2014). Do our patients have enough to eat? Food insecurity among urban low-income cancer patients. *Journal of Health Care for the Poor and Underserved*. https://doi.org/10.1353/hpu.2014.0145

Gijsen, R., Hoeymans, N., Schellevis, F. G., Ruwaard, D., Satariano, W. A., & Van Den Bos, G. A. M. (2001). Causes and consequences of comorbidity: A review. *Journal of Clinical Epidemiology*. https://doi.org/10.1016/S0895-4356(00)00363-2

González-Fernández, D., Pons, E. del C., Rueda, D., Sinisterra, O. T., Murillo, E., Scott, M. E., & Koski, K. G. (2017). C-reactive protein is differentially modulated by co-existing infections, vitamin deficiencies and maternal factors in pregnant and lactating indigenous Panamanian women. *Infectious Diseases of Poverty*. https://doi.org/10.1186/s40249-017-0307-1

Gowda, C., Hadley, C., & Aiello, A. E. (2012). The association between food insecurity and inflammation in the US adult population. *American Journal of Public Health*, *102*(8), 1579–1586. https://doi.org/10.2105/AJPH.2011.300551

Gubert, M. B., Spaniol, A. M., Bortolini, G. A., & Pérez-Escamilla, R. (2016). Household food insecurity, nutritional status and morbidity in Brazilian children. *Public Health Nutrition*. https://doi.org/10.1017/S1368980016000239

Gucciardi, E., Vogt, J. A., DeMelo, M., & Stewart, D. E. (2009). Exploration of the relationship between household food insecurity and diabetes in Canada. *Diabetes Care*. https://doi.org/10.2337/dc09-0823

Hill, C. M., Tseng, A. S., Holzhauer, K., Littman, A. J., & Jones-Smith, J. C. (2022). Association between health care access and food insecurity among lower-income older adults with multiple chronic conditions in Washington State, United States. *Public Health Nutrition*, (Mcc), 1–9. https://doi.org/10.1017/S1368980022001240

Jafari, F., Ehsani, S., Nadjarzadeh, A., Esmaillzadeh, A., Noori-Shadkam, M., & Salehi-Abargouei, A. (2017). Household food insecurity is associated with abdominal but not

general obesity among Iranian children. *BMC Public Health*. https://doi.org/10.1186/s12889-017-4262-3

Jih, J., Nguyen, T. T., Jin, C., Seligman, H., Boscardin, W. J., & Ritchie, C. S. (2020). Food Insecurity Is Associated with Behavioral Health Diagnosis Among Older Primary Care Patients with Multiple Chronic Conditions. *Journal of General Internal Medicine*. https://doi.org/10.1007/s11606-019-05569-4

Jih, J., Stijacic-Cenzer, I., Seligman, H. K., Boscardin, W. J., Nguyen, T. T., & Ritchie, C. S. (2018). Chronic disease burden predicts food insecurity among older adults. *Public Health Nutrition*. https://doi.org/10.1017/S1368980017004062

Kazancioğlu, R. (2013). Risk factors for chronic kidney disease: An update. In *Kidney International Supplements*. https://doi.org/10.1038/kisup.2013.79

Koné Pefoyo, A. J., Bronskill, S. E., Gruneir, A., Calzavara, A., Thavorn, K., Petrosyan, Y., Maxwell, C. J., Bai, Y., & Wodchis, W. P. (2015). The increasing burden and complexity of multimorbidity. *BMC Public Health*. https://doi.org/10.1186/s12889-015-1733-2

Leung, C. W., Kullgren, J. T., Malani, P. N., Singer, D. C., Kirch, M., Solway, E., & Wolfson, J. A. (2020). Food insecurity is associated with multiple chronic conditions and physical health status among older US adults. *Preventive Medicine Reports*. https://doi.org/10.1016/j.pmedr.2020.101211

McPhail, S. M. (2016). Multimorbidity in chronic disease: Impact on health care resources and costs. *Risk Management and Healthcare Policy*. https://doi.org/10.2147/RMHP.S97248

Mendis, S., Puska, P., & Norrving, B. (2011). Global atlas on cardiovascular disease prevention and control. *World Health Organization*.

Mendy, V. L., Vargas, R., Cannon-Smith, G., Payton, M., Enkhmaa, B., & Zhang, L. (2018).

Food insecurity and cardiovascular disease risk factors among Mississippi adults. *International Journal of Environmental Research and Public Health*. https://doi.org/10.3390/ijerph15092016

Menotti, A., Mulder, I., Nissinen, A., Giampaoli, S., Feskens, E. J. M., & Kromhout, D. (2001). Prevalence of morbidity and multimorbidity in elderly male populations and their impact on 10-year all-cause mortalitym: The FINE study (Finland, Italy, Netherlands, elderly). *Journal of Clinical Epidemiology*. https://doi.org/10.1016/S0895-4356(00)00368-1

Modesti, P. A., Reboldi, G., Cappuccio, F. P., Agyemang, C., Remuzzi, G., Rapi, S., Perruolo, E., Parati, G., Agostoni, P., Barros, H., Basu, S., Benetos, A., Ceriello, A., Prato, S. Del, Kalyesubula, R., Kilama, M. O., O'Brien, E., Perlini, S., Picano, E., Redon, J., Stuckler, D., van Bortel, L. M., Zhao, D., Bamoshmoosh, M., Bennet, L., Bruno, R. M., Carlsson, A. C., Cifkova, R., Fadnes, L. T., Grech, H., Klocek, M., Kumar, B., Lalic, N., Manolis, A. J., Nørredam, M., Massetti, L., de Courten, M. P., Pereira, M., Pratali, L., Siegert, A., Szklarska, A., Tendera, M., Twagirumukiza, M., Volodina, A., Watfa, G., Karaye, K. M., Phanzu, B. K., Dzudie, A., N'Guetta, R., Kiiza, M. C., Gudina, E. K., Longo-Mbenza, B., Mucumbitsi, J., Anisiuba, B., Ibrahim, T. A., & Okechukwu, O. S. (2016). Panethnic differences in blood pressure in Europe: A systematic review and meta-analysis. *PLoS ONE*. https://doi.org/10.1371/journal.pone.0147601

Moher, D., Liberati, A., Tetzlaff, J., Altman, D. G., Altman, D., Antes, G., Atkins, D., Barbour, V., Barrowman, N., Berlin, J. A., Clark, J., Clarke, M., Cook, D., D'Amico, R., Deeks, J. J., Devereaux, P. J., Dickersin, K., Egger, M., Ernst, E., Gøtzsche, P. C., Grimshaw, J., Guyatt, G., Higgins, J., Ioannidis, J. P. A., Kleijnen, J., Lang, T., Magrini, N., McNamee, D., Moja, L., Mulrow, C., Napoli, M., Oxman, A., Pham, B., Rennie, D., Sampson, M., Schulz, K. F.,

Shekelle, P. G., Tovey, D., & Tugwell, P. (2009). Preferred reporting items for systematic reviews and meta-analyses: The PRISMA statement. *PLoS Medicine*. https://doi.org/10.1371/journal.pmed.1000097

Moradi, S., Arghavani, H., Issah, A., Mohammadi, H., & Mirzaei, K. (2018). Food insecurity and anaemia risk: A systematic review and meta-analysis. *Public Health Nutrition*. https://doi.org/10.1017/S1368980018001775

Nagel, G., Peter, R., Braig, S., Hermann, S., Rohrmann, S., & Linseisen, J. (2008). The impact of education on risk factors and the occurrence of multimorbidity in the EPIC-Heidelberg cohort. *BMC Public Health*. https://doi.org/10.1186/1471-2458-8-384

Nunes, B. P., Flores, T. R., Mielke, G. I., Thumé, E., & Facchini, L. A. (2016). Multimorbidity and mortality in older adults: A systematic review and meta-analysis. *Archives of Gerontology and Geriatrics*. https://doi.org/10.1016/j.archger.2016.07.008

O'neal, L. J., Jo, A., Scarton, L., & Bruce, M. A. (2022). Food Insecurity Is Associated with Mental–Physical Comorbidities among U.S. Adults: NHANES 2013 to 2016. *International Journal of Environmental Research and Public Health*. https://doi.org/10.3390/ijerph19031672

Oh, H., Smith, L., Jacob, L., Du, J., Shin, J. Il, Zhou, S., & Koyanagi, A. (2022). Food insecurity and mental health among young adult college students in the United States. *Journal of Affective Disorders*. https://doi.org/10.1016/j.jad.2022.02.009

Palakshappa, D., Speiser, J. L., Rosenthal, G. E., & Vitolins, M. Z. (2019). Food Insecurity Is Associated with an Increased Prevalence of Comorbid Medical Conditions in Obese Adults: NHANES 2007–2014. *Journal of General Internal Medicine*. https://doi.org/10.1007/s11606-019-05081-9

Pathirana, T. I., & Jackson, C. A. (2018). Socioeconomic status and multimorbidity: a systematic review and meta-analysis. *Australian and New Zealand Journal of Public Health*. https://doi.org/10.1111/1753-6405.12762

Rosas, L. G., Guendelman, S., Harley, K., Fernald, L. C. H., Neufeld, L., Mejia, F., & Eskenazi, B. (2011). Factors associated with overweight and obesity among children of Mexican descent: Results of a binational study. *Journal of Immigrant and Minority Health*. https://doi.org/10.1007/s10903-010-9332-x

Safaei, M., Sundararajan, E. A., Driss, M., Boulila, W., & Shapi'i, A. (2021). A systematic literature review on obesity: Understanding the causes & consequences of obesity and reviewing various machine learning approaches used to predict obesity. *Computers in Biology and Medicine*. https://doi.org/10.1016/j.compbiomed.2021.104754

Seligman, H. K., Tschann, J., Jacobs, E. A., Fernandez, A., & López, A. (2012). Food insecurity and glycemic control among low-income patients with type 2 diabetes. *Diabetes Care*. https://doi.org/10.2337/dc11-1627

Sharkey, J. R. (2003). Risk and Presence of Food Insufficiency Are Associated with Low Nutrient Intakes and Multimorbidity among Homebound Older Women Who Receive Home-Delivered Meals. In *Journal of Nutrition*. https://doi.org/10.1093/jn/133.11.3485

Smith, L., Il, J., Louis, S., López, G. F., Felipe, S., Mark, S., & Hans, A. T. (2022). Food insecurity and physical multimorbidity among adults aged $\geq$ 50 years from six low‑ and middle‑ income countries. *European Journal of Nutrition*, (0123456789). https://doi.org/10.1007/s00394-022-02999-5

Summit, W. F. (1996). Rome Declaration on World Food Security and World Food Summit Plan of Action Rome Declaration on World Food Security. *World Food Summit*.

Tarasuk, V., Mitchell, A., McLaren, L., & McIntyre, L. (2013). Chronic physical and mental health conditions among adults may increase vulnerability to household food insecurity. *Journal of Nutrition*. https://doi.org/10.3945/jn.113.178483

Valtorta, N. K., & Hanratty, B. (2013). Socioeconomic variation in the financial consequences of ill health for older people with chronic diseases: A systematic review. *Maturitas*. https://doi.org/10.1016/j.maturitas.2013.01.015

Violan, C., Foguet-Boreu, Q., Flores-Mateo, G., Salisbury, C., Blom, J., Freitag, M., Glynn, L., Muth, C., & Valderas, J. M. (2014). Prevalence, determinants and patterns of multimorbidity in primary care: A systematic review of observational studies. *PLoS ONE*. https://doi.org/10.1371/journal.pone.0102149

Violán, C., Foguet-Boreu, Q., Hermosilla-Pérez, E., Valderas, J. M., Bolíbar, B., Fàbregas-Escurriola, M., Brugulat-Guiteras, P., & Muñoz-Pérez, M. Á. (2013). Comparison of the information provided by electronic health records data and a population health survey to estimate prevalence of selected health conditions and multimorbidity. *BMC Public Health*. https://doi.org/10.1186/1471-2458-13-251

Vozoris, N. T., & Tarasuk, V. S. (2003). Household food insufficiency is associated with poorer health. *Journal of Nutrition*. https://doi.org/10.1093/jn/133.1.120

World Health Organization. (2018). Ageing and health.

Zhou, A., & Hyppönen, E. (2022). Vitamin D deficiency and C-reactive protein: a bidirectional Mendelian randomization study. *International Journal of Epidemiology*, 1–12. https://doi.org/10.1093/ije/dyac087

Supporting information

S1 Table. **PRISMA checklist**

S1 Fig. Funnel plot in the meta-analysis on the association between food insecurity and multimorbidity.

S2 Fig. Funnel plot in the meta-analysis on the association between multimorbidity and food insecurity.

Table 1. Characteristics of the observational studies.

Studies defined multimorbidity as the outcome of interest

Author / Year Country	Study Design population	Main measures	Multimorbidity definition / occurrence	Food insecurity definition / occurrence	Comparison group	Multimorbidity assessment	Food Insecurity assessment	Confounders	Main findings
Leung et al, 2020 U.S.	Cross- sectional study N = 2048 50–80 y	FI Multiple chronic conditions	NR / 94,4%	>2 affirmative responses from 6-item short form US Household Food Security Survey Module / 14%	0 – 1 condition	Self-reported diagnosis	6-item U.S. Household Food Security Survey Module	age, sex, race ethnicity, educational attainment, marital status, annual, household income, employment health insurance	FI was associated with multimorbidity. 2 -3 chronic conditions: (RRR 1.60, 95% CI 1.08, 2.36) ≥ 4 chronic conditions (RRR 2.59, 95% CI 1.55, 4.33)
Sharkey R. Joseph 2003 U.S.	Cross- sectional study N = 279 Women >60 y Home-delivered meal recipients	FI Multimorbidity	≥3 disease conditions / 47,3%	Responded yes, to either absence of food situation / 10%	<3 disease conditions	Self-reported diagnosis	Four self-reported FI risk situations questions	Race, income, education, age, living arrangement, marital status, tobacco use, Food Stamp, Program participation, IADL, BMI	FI was associated with greater than three times higher odds of multimorbidity (OR:3.69, 95% CI 1.14 – 12.00)

Smith et al, 2022 China Ghana India Mexico Russia South Africa	Cross- sectional study N = 34 129 > 50 y	FI Multimorbidity	≥2 chronic conditions / 45.5 %	Positive answers in two questions / 11.8%	< 2 chronic conditions	Self-reported diagnosis	Two questions about FI	Country, age, sex, education, wealth	FI was associated with multimorbidity (OR:1.40, 95% CI 1.15 – 1.70)
O'Neal et al, 2022 US	Cross- sectional study N = 8 948 > 18 y	FI chronic conditions	≥ 1 chronic cardiometabolic condition and depression / 49.2 %	>1 affirmative responses from 18-item form US Household Food Security Survey Module / 25.1%	0 chronic conditions	Self-reported diagnosis	18-item US Household Food Security Survey Module	Age, sex, and race, ethnicity	FI was associated with multimorbidity. Very low FI: OR: 2.4 (95% CI 1.06 –5.43) Low FI: OR: 2.2 (95% CI 1.23 –3.95) Marginal FI: OR: 3.6 (95% CI 2.26 –5.76)

Studies defined food insecurity as the outcome of interest

Author / Year Country	Study Design population	Main measures	Multimorbidity definition / occurrence	Food insecurity definition / occurrence	Comparison group	Multimorbidity assessment	Food Insecurity assessment	Confounders	Main findings
Jih et al, 2018 U.S.	Cross- sectional study N = 3552 > 50 y Annual household income < 300% of the FPL	FI Multiple chronic conditions	NR / 83,5%	>2 affirmative responses from 6-item short form US Household Food	0 – 1 affirmative responses	Self-reported diagnosis	6-Item U.S. Household Food Security Survey Module	Age, sex, race, ethnicity, marital status, health insurance status, self-rated health,	Multimorbidity was associated with FI. 2 -4 chronic conditions: (OR 2.12,

				Security Survey Module / 27.8%				employment status, household size, education, wealth, household size, smoking status and BMI	95% CI 1.45, 3.09)<br>> 4 chronic conditions (OR 3.64, 95% CI 2.47, 5.37)
Tarasuk et al, 2013 Canada	Cross- sectional study N = 77053 18–64 y	FI Multimorbidity	NR	1 affirmative response from18-item US Household Food Security Survey Module / 11.8%	0 affirmative responses	Self-reported diagnosis	18-item US Household Food Security Survey Module	household sociodemographic.	Multimorbidity was associated with FI.<br>1 chronic condition: OR: 1.43 (95% CI 1.28 – 1.59)<br>2 chronic conditions: OR:1.86 (95% CI 1.62–2.14)<br>3 chronic conditions: OR: 3.44 (95% CI 3.02–3.93)
Jih et al, 2020 U.S.	Cross- sectional study N = 475 > 60 y	FI Multiple chronic conditions	≥ 2 chronic conditions / 98,3%	3+ affirmative responses from 10-item U.S. Adult Food Security Survey Module / 8.2%	NR	Diagnoses from the Elixhauser Comorbidity Index	10-item U.S. Adult Food Security Survey Module	Age, sex, race ethnicity, presence of behavioral health diagnosis, multiple chronic conditions, BMI, number of primary care visits in	Multimorbidity was associated with FI.<br>≥1 chronic conditions: OR: 4.1 (95% CI 1.9 – 9.0)<br>≥5 chronic conditions: OR: 1.84

								the last year that were significantly different between older adults with and without FI	(95% CI 0.78 – 4.3)
Fernandes et al, 2018 Portugal	Cross- sectional study N = 1885 > 65 y	FI Multiple chronic conditions	NR	1 affirmative response from Psychometric scale / 23%	0 affirmative response	Self-reported diagnosis	Psychometric scale adapted from the Brazilian Insecurity Scale	Age, gender, education level, health region	The presence of ≥ 1 chronic conditions was associated with FI OR: 1.161 (95% CI 1.158 – 1.164)

Abbreviations: N: Sample, FPL: Federal Poverty Level, FI: Food Insecurity, U.S.: United States, NR: Not Reported, BMI: Body Mass Index, IADL: Instrumental Activities of Daily Living, OR: Odd Ratio, AOR: Adjusted Odd Ratio

Table 2. Quality assessment using Newcastle-Ottawa scale adapted for cross-sectional studies.

				Studies defined multimorbidity as the outcome of interest					
**Author, year**	**Selection**				**Comparability**	**Outcome**		**Total quality score**	**Results**
	(1) Representativeness of the sample	(2) Sample size	(3) Non responders	(4) Ascertainment of the exposure	(1) Comparability of subjects on the basis of the design or analysis	(1) Assessment of outcome	(2) Statistical test		
Leung et al, 2020	*	-	*	-	**	-	*	5/10	Satisfactory
Sharkey R. 2003	*	-	*	-	**	-	*	5/10	Satisfactory
Smith et al, 2022	*	-	*	-	**	-	*	5/10	Satisfactory
O'Neal et al, 2022	*	-	*	-	**	-	*	5/10	Satisfactory
				Studies defined food insecurity as the outcome of interest					
**Author, year**	**Selection**	**Comparability**	**Outcome**	**Total quality score**					
	(1) Representativeness of the sample	(2) Sample size	(3) Non responders	(4) Ascertainment of the exposure	(1) Comparability of subjects on the basis of the design or analysis	(1) Assessment of outcome	(2) Statistical test		

Jih et al, 2018	-	-	*	-	**	-	*	4/10	Unsatisfactory
Tarasuk et al, 2013	*	-	*	-	**	-	*	5/10	Satisfactory
Jih et al, 2020	-	-	-	*	**	-	*	4/10	Unsatisfactory
Fernandes et al, 2018	*	-	*	-	**	-	*	5/10	Satisfactory

Abbreviations:
A maximum of 5 stars can be awarded for the selection.
A maximum of 2 stars can be awarded for the comparability.
A maximum of 3 stars can be awarded for the outcome.

Figure 1

Click here to access/download;Figure;Figure 1.docx

Fig 1. PRISMA inclusion flowchart

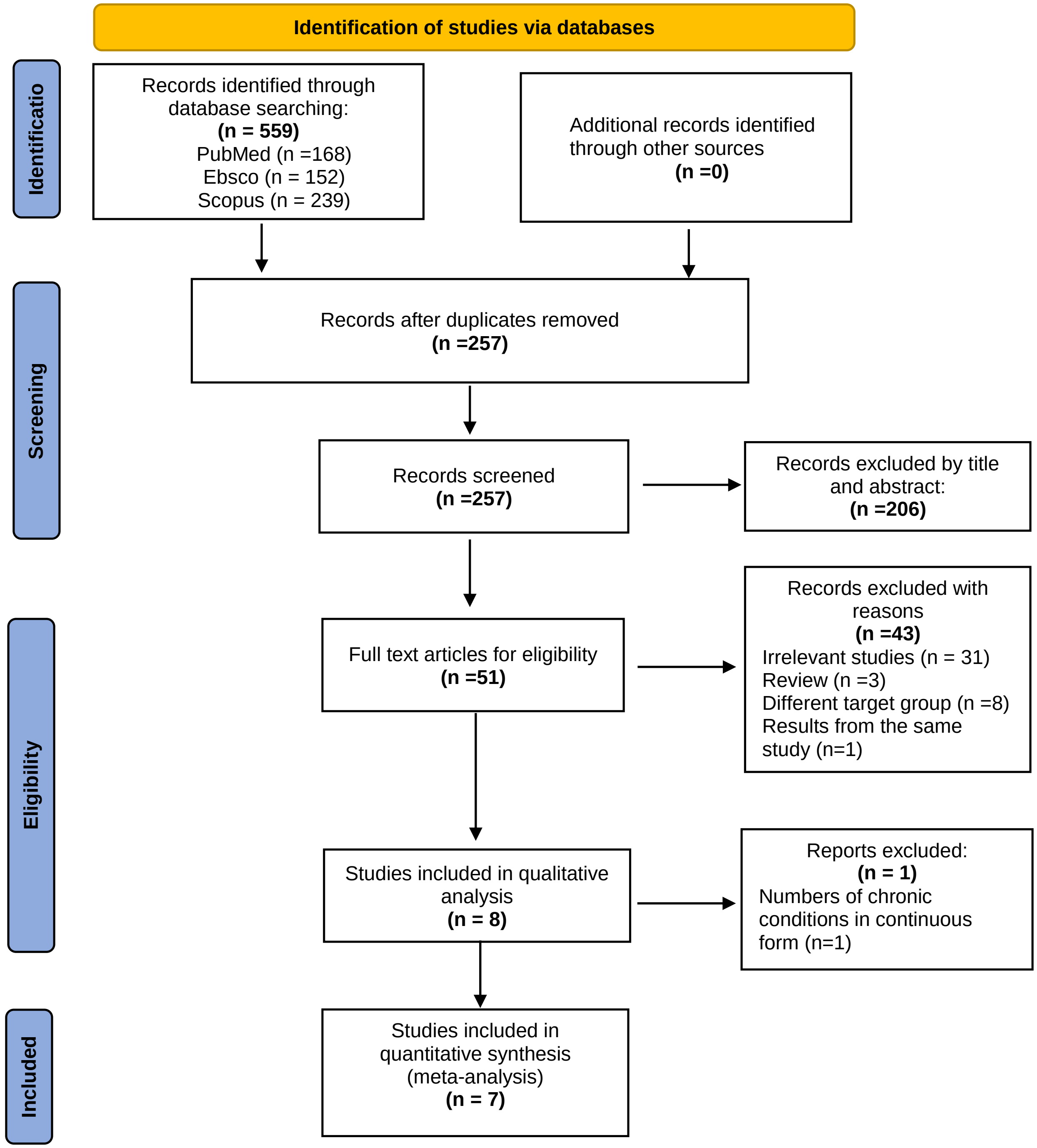

 Click here to access/download;Figure;Figure 2.docx

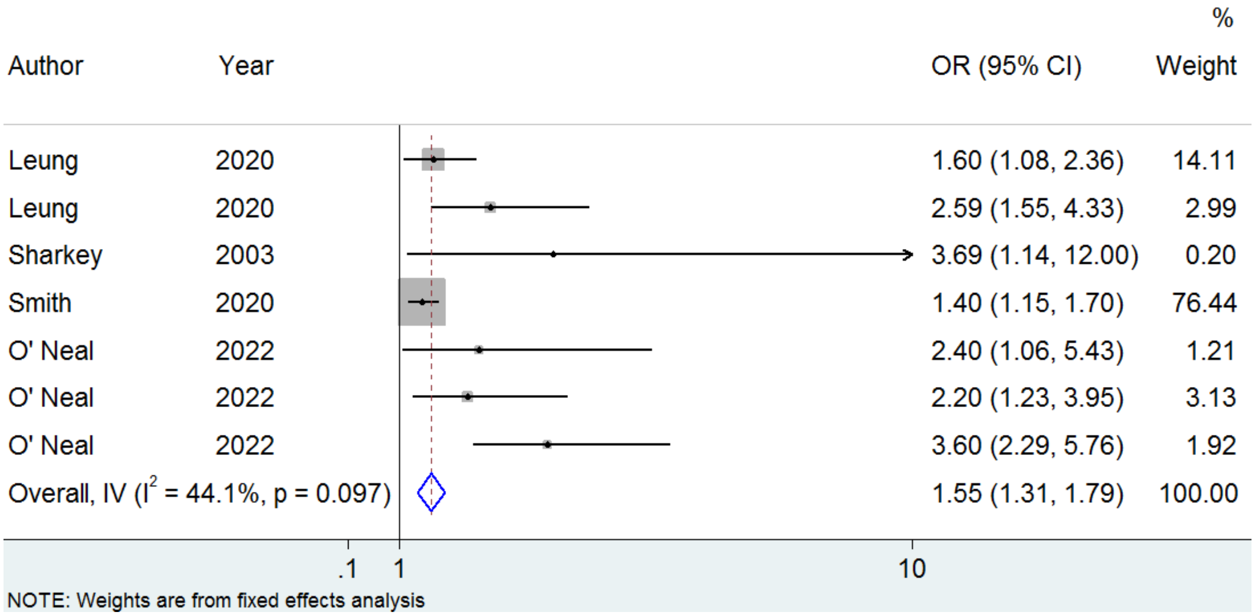

Fig 2. Forest plot for the meta-analysis for the association between food insecurity and multimorbidity.

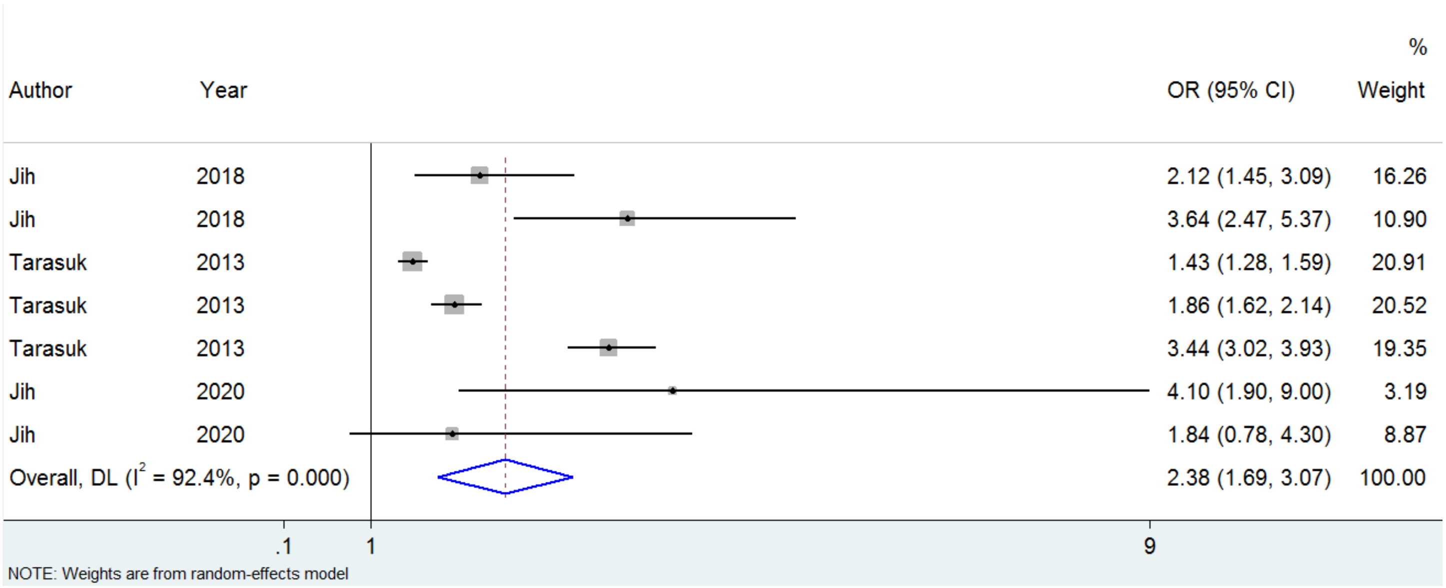

Fig 3. Forest plot for the meta-analysis for the association between multimorbidity and food insecurity.

Click here to access/download
Supporting Information
S1_Figure.docx

Click here to access/download
Supporting Information
S1_Table.docx

Click here to access/download
Supporting Information
S2_Figure.docx

Click here to access/download
Supporting Information
S2_Table.docx
