# Supplementary material for: Multimorbidity and food insecurity in adults: A systematic review and meta-analysis
Source: PLoS One. 2023 Jul 6;18(7):e0288063. doi: 10.1371/journal.pone.0288063 (PMC10325088; doi:10.1371/journal.pone.0288063)
Supplement: Attachment — Submitted filename: Evaluation Report - Manuscript Review Assignment for PONE-D-22-34406 is due soon.docx [file pone.0288063.s005.docx]

# **EVALUATION REPORT**

## **Study Title**: Food Insecurity and multimorbidity in adult population: A systematic review and Meta-analysis

## 1. **Please summarise the main findings of the study and its purpose**

This systematic review and meta-analysis’s objectives are essential in understanding the existing evidence of the relationship between food insecurity and adult multimorbidity. The research topic is significant, considering the older adult population’s unprecedented growth and the projected increase in the prevalence of multimorbidity and its relationship with several driving factors, such as food insecurity associated with multimorbidity globally.

Thus, it is imperative to map the existing evidence on food insecurity and adult multimorbidity to summarise what is known about the prevalence, incidence and association to support the possible reverse causation between them. Here, Maria Kantilafti *et al*., outline a review that examines the existing evidence.‬‬

## 2. **Please highlight the limitations and advantages**

This study has many strengths, such as clear objectives and the focus on a specific population age group. However, overall the study includes key elements of a systematic review and meta-analysis plan but requires additional attention concerning existing literature and methodological details, such as specifying the guidelines of the method used to design the systematic review component and meta-analysis component, also approaches used to present and synthesise results. I provide further explanations below.

## 3. **Are there objective errors or fundamental flaws? If yes, please detail your concerns**

I have offered specific comments below. Thus, improving the clarity of the study’s design methodology will, in my mind, undoubtedly increase its impact. The study design methodology is currently missing very vital information that should be pre-specified and as far as possible in the Abstract and Methodology sections:

### **ABSTRACT**

=========

#### **Major comments:**

1) The authors have selected an essential topic for the study. As applicable in the title and abstract, the authors must provide an informative and balanced summary of what was done and the findings in the abstract section.
a) I, therefore, ask the authors to please ***Report an abstract addressing each item in the PRISMA 2020 for Abstracts checklist*** (A link to the checklist is shared below with the authors)

<https://view.officeapps.live.com/op/view.aspx?src=http%3A%2F%2Fwww.prisma-statement.org%2Fdocuments%2FPRISMA_2020_abstract_checklist.docx&wdOrigin=BROWSELINK>.

#### **Minor comment:**

2) Please revise the conclusion in the abstract to avoid overly casual language.
3) Specify the primary source of funding for the review.
4) Provide the register name and registration number; as such, detail is warranted in the methodology section and also briefly here.

### **INTRODUCTION**

==============

#### **Major comments:**

5) The rationale for undertaking this review study in the context of already existing evidence has not been clearly explained. I am uncertain about the novelty of the research question and the rationale for the investigation being reported.
b) I suggest that the authors may also consider reporting how the research question is built on existing evidence; the reader needs to understand these links.
a) The last paragraph needs to have a statement about what is unknown and how this study adds to such knowledge.

#### **Minor comment:**

6) The authors have not clearly explained effort to avoid duplication and identify critical reviews comparable to their current project. I suggest that:
a) The authors may also consider conducting a preliminary search for published systematic reviews or protocols in PROSPERO, MEDLINE, Cochrane Database of Systematic Reviews and the Joanna Briggs Institute (JBI) Evidence synthesis.
b) However, if other reviews addressing the same (or a largely similar) question are available, explain why the current review was considered necessary and how this study adds to such knowledge.
c) Also, including a short description/discussion of the preliminary search result is warranted briefly here, or make use of diagrams to illustrate publication interest or lack thereof over the years.

### **METHODS**

========

#### **Major comments:**

7) Please specify the Protocol Register name, registration number, and the link to the protocol which informed the design and conduct of the review reported here.
8) The authors do not provide an explicit statement of questions being addressed or explain how the research question’s eligibility for a systematic review project was determined. It should be explicitly mentioned with reference to participants and each element of interest.
a) To identify the research question’s eligibility for a systematic review project, the authors must clearly state all objective(s) and question(s) the review addresses. Please note that these two needs to be expressed in terms of the relevant question formulation framework recommended according to the JBI Manual for Evidence Synthesis.
9) The authors must provide an informative report of the methodology section. I, therefore, ask the authors to please report a methodology section addressing each item in the PRISMA 2020 expanded checklist (A link to the checklist is shared below). The current section lacks details:

<http://www.prisma-statement.org/documents/PRISMA_2020_expanded_checklist.pdf>
